# Cholecystocolonic fistula in exacerbated chronic cholecystitis: A case report

**DOI:** 10.1016/j.radcr.2023.10.060

**Published:** 2023-12-03

**Authors:** Francesco Manti, Giuliana Conti, Caterina Battaglia, Cesare Oliveti, Angela Commisso, Marco Giurdanella, Domenico Laganà

**Affiliations:** Radiology Unit, Department of Experimental and Clinical Medicine, “Magna Graecia” University, Catanzaro, Italy

**Keywords:** Cholecystocolonic fistula, Biliary disease, T2w and SPAIR-MRI, WBCT

## Abstract

The cholecystocolonic fistula (CCF) is an atypical variant of biliary disease, and it is the second most common intestinal fistula after cholecystoduodenal fistula. Intraoperative diagnosis is frequent, which implies challenging surgical management, especially in patients, often aged, with comorbidities. The rarity of this condition, atypical and various presentation, diagnostic and management complexity, makes it a unique surgical entity. We report our experience of an 84-year-old man with a history of chronic cholecystitis who presented with nonspecific symptoms. The imaging tests aroused the suspicion of gallbladder-colic fistula in the preoperative diagnosis, facilitating the subsequent surgical treatment that confirmed the diagnosis.

## Introduction

Biliary fistulas to various structures, especially to the gastrointestinal tract (internal fistulas) and the abdominal wall (external fistulas), have been disclosed in the literature. Particularly, the cholecystocolonic fistula (CCF) is a late complication of chronic gallbladder disease. It represents the second most common gallbladder-enteric fistula (8%-26.5% of gallbladder-enteric fistulas) after cholecystoduodenal fistula. Its incidence is approximately 0.06%-0.14% of patients undergoing cholecystectomy, and more than 90% of these cases are diagnosed intraoperatively [1], [2], [3]. The presentation is usually atypical, with mild and nonspecific symptoms, diagnostic challenges, and complex surgical management [2,3]. Besides intraoperative failure, it can lead to surgical morbidities like colonic perforation, peritonitis, or death. Therefore, a timely diagnosis by clinical presentation and preoperative assessment are important, resulting in successful surgical management.

## Case report

**Fig. 1:**
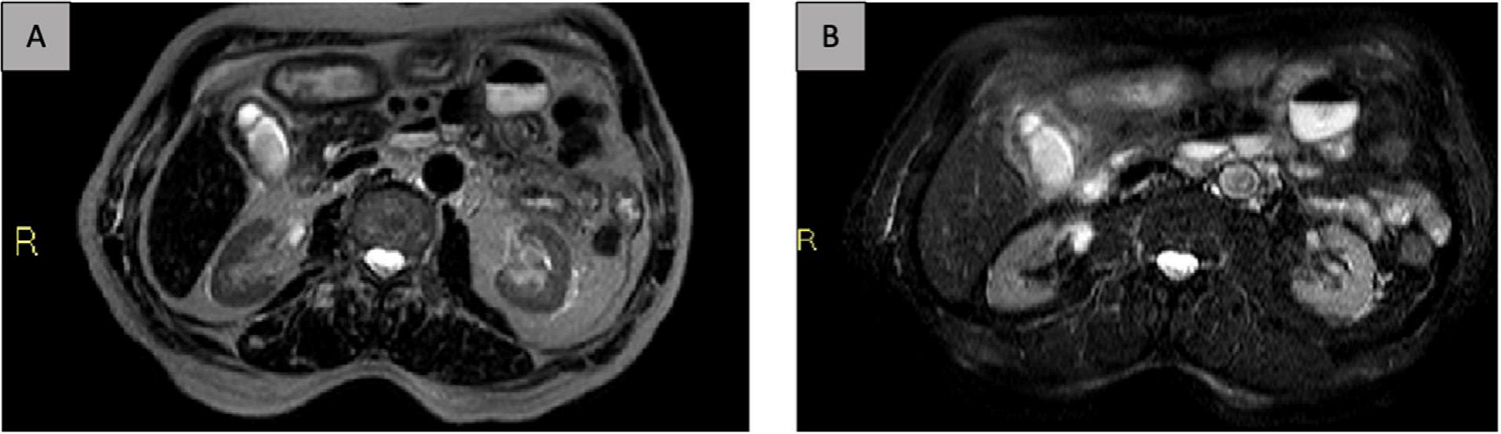
Axial MR image of the abdomen (A. T2w, B. SPAIR) demonstrated fluid collection in the gallbladder bed in a chronic inflammation background it.

An 84-year-old male came to our department reporting the onset of upper abdominal pain for several days and an on-and-off fever for three weeks. On clinical examination, the abdomen was soft and symmetric, both deep and superficial palpation, detected mild tenderness in the right hypochondrium, without jaundice or positivity of Murphy's sign. There were no visible lesions or scars. The aorta was midline without bruit or visible pulsation. Umbilicus was midline without herniation. Bowel sounds were present and normoactive in all four quadrants. No masses, hepatomegaly, or splenomegaly were noted. The patient's past medical history was significant for an episode of acute cholecystitis that occurred a month before and was treated with conservative treatment which consisted of fasting from food and fluids to put the gallbladder at rest during the most acute phase of inflammation. On that occasion, intravenous fluids were administered, and an elective surgery was planned. Patient was admitted and investigated with a provisional diagnosis of gallstone disease. It is essential to mention that the patient was affected by hypertension, diabetes mellitus type 2, obesity of the 2nd degree (BMI of 32 Kg/m^2^), dyslipidemia, smoking, CAD, and biliary lithiasis. In our ward, the patient underwent a magnetic resonance cholangiopancreatography (MRCP), which showed the presence of an essential inflammatory process affecting the gallbladder with a fluid layer in the peri-bladder area (Fig. 1).

He was hospitalized, and vital signs were routinely checked and held stable. Laboratory evaluation (Table 1) showed the presence of neutrophilic leukocytosis, mild hypochromic microcytic anemia, modest increase in transaminases, gamma-GT and direct bilirubin.

**Table 1:** Laboratory examinations at hospitalization.

	Patient value	Normal value
Alanine aminotransferase (ALT) U/L	270	< 31
Aspartate aminotransferase (AST) U/L	130	< 32
Gamma – glutamyltransferase (GGT) U/L	116	5 - 36
Direct bilirubin mg/dL	3.14	0.00-0.30

**Fig. 2:**
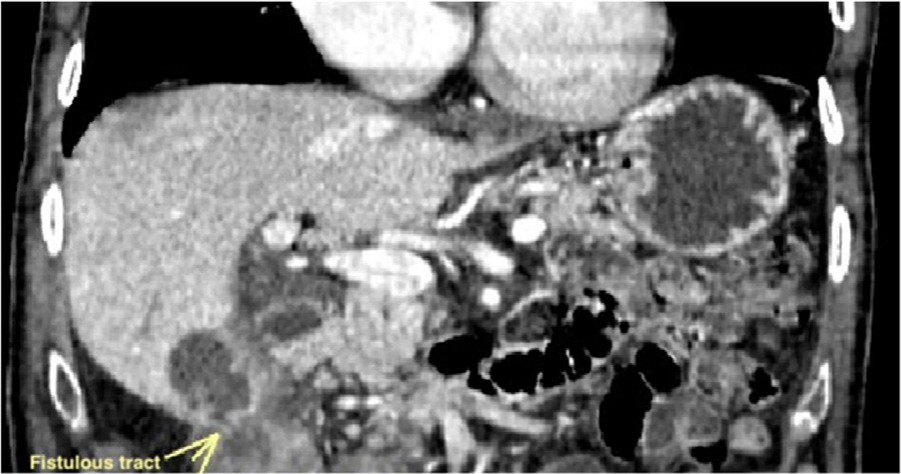
Coronal CT image shows a close approximation of hepatic flexure of colon with the gallbladder which suggests a fistulous communication (arrow).

The supine abdominal radiograph was negative for pneumobilia or intestinal obstruction, while ultrasonography was characterized by chronic gallbladder disease with a fluid flap in the gallbladder bed (images not shown). Moreover, biphasic contrast medium injection in whole-body computed tomography (WBCT) was assessed. It revealed thickened gallbladder wall with multiple calculi embedded within it and, in the fundal region, a slight soft tissue tract leading to the adherent adjacent part of the hepatic flexure of the colon, which showed thickened and anatomically uneven walls as well (Fig. 2). An area of inflammatory reaction between the hepatic flexure and the gallbladder coexisted and a fair amount of peri-bladder and peri-colic outflow together with the inhomogeneous aspect of the adjacent mesenteric tissue was seen with reactive lymphadenopathy. The rest of the abdomen was unremarkable: no signs of bowel obstruction or images of calcific-density stones in the sigmoid colon were present.

Based on the radiological findings, fistulous communication between the fundal region of the gallbladder and the hepatic flexure of the colon was diagnosed.

The patient underwent laparotomic treatment of the gallbladder and the hepatic flexure of the colon. Then, he was followed up over 9 days on inpatient care basis and discharged with a complete resolution of the clinical picture.

## Discussion

CCFs are one of the rare complications associated with gallstone disease. A persistent inflammatory process can lead to ulceration and ischemia of gallbladder walls and closed organs due to the erosive and fistulization process. In addition to a chronic inflammatory process, previous surgical treatments represent other possible causes, in particular, gastric surgery [4], cholecystectomy [5], and abdominal traumatic or iatrogenic injury [6,7].

Symptomatology comprises a broad spectrum, varying from diarrhea, which is the most frequent symptom (70%) [8], [9], [10]–11,14], and other possible symptoms such as abdominal distension with pain in the right hypochondrium, fever, and jaundice (as a potential case of cholangitis) [12,13].

Another uncommon but described presentation of the CCF is acute onset complications like gallstone ileus, a stone located in the sigmoid colon after perforation of the walls, and massive bleeding or liver abscess [15]. Finally, an association between gallbladder-colic fistula and gallbladder carcinoma has been reported.

This report describes a case where abdominal pain and fever were the only symptoms. The patient's medical history was decisive in arousing the suspicion of this rare complication.

Even if the patient's clinical situation was stable, laboratory exams showed an increase in the selected cholestasis indices, associated with transaminases as hepatic functional evaluation, requiring further investigation by imaging tests.

Generally, the diagnosis of CCF is achieved preoperatively in only 8% of cases. As a first approach, US evaluation offers prompt and rapid diagnosis of chronic cholecystitis, but no obvious signs of a fistula may be present. In most cases, it is useful to screen the abdomen. In fact, ultrasound is not usually diagnostic, while the survey with barium enema has sometimes allowed the identification of the cholecystocolic fistula [17], although it is burdened by false negatives [4,^10^,^13^,^16^,18]. Then, as some studies show, the presence or absence of pneumobilia can be confirmed by using contrast CT or radiographic investigation [4,16]. MRI or MRCP can also be done to diagnose intrahepatic pneumobilia. The presence of pneumobilia would raise the suspicion of a fistula. Endoscopic ultrasound (EUS) can be employed to confirm the presence of a gallbladder-colon fistula, but at the same time, CT or MRCP can be performed noninvasively. It is crucial to identify the cause of the fistula which could be due to gallstones. Indeed, inflammatory conditions and neoplasia should be excluded by performing CT or ERCP. The results of endoscopic retrograde cholangiopancreatography (ERCP) are also variable, allowing diagnosis in some cases [4,^10^,^17^,19], but not in others [12,^13^,^17^,18]. Preoperative diagnosis is also problematic, even with computed tomography (CT) [22,23], MRI [24], and echo-endoscopy [18]. Finally, some authors reported the usefulness of intraoperative cholecystography for the definitive diagnosis of gallbladder-colic fistula [12] and for identifying gallstones in the common bile duct.

In this medical case, biphasic contrast medium injection in whole-body computed tomography (WBCT) was able to detect fistulous communication between the fundal region of the gallbladder and the hepatic flexure of the colon, together with chronic cholecystitis. An abdominal CT scan is the gold standard imaging method with a sensitivity and specificity of 93% and 100%, respectively [26]. The CT scan is useful to detect the size, the number as well as the morphology of the gallstones, and to identify secondary findings such as mechanical ileus, biliary-enteric fistulas, or the presence of air in the gallbladder [27]. In addition, colonoscopy is rarely applied [16,20] as well as scintigraphy [10,21].

According to the literature, the preoperative diagnosis of CCF is quite challenging. The absence of preoperative diagnosis often forces a more complex procedure due to the intraoperative diagnosis, changing the surgical options from an elective course to an emergency one, which usually involves adhesiolysis and bowel resection too, as main complications, especially in aged and comorbid patients. Alternatively, when it is possible, it is warmly suggested ERCP with endoscopic sphincterotomy as the primary potential option (which also allows the removal of any gallstones), reducing the pressure in the biliary ducts and promoting the closure of the uncomplicated fistula.

Several surgical treatment approaches have been reported for both uncomplicated gallbladder-colic fistula and those associated with complications. However, no “consensus” has been developed to address the optimal surgical management of CCF. Conservative management is also suggested for uncomplicated fistulas in frail patients with limited life expectancy [25].

From our experience with this case, we tend to disagree with the conservative management suggested by literature, particularly for frail patients like ours. As there is evidence of biliary obstruction risk with potential biliary sepsis from colonic bacteria translocation or of the passage of gallstone in the sigmoid colon, all with deleterious physiologic changes and unfavorable consequences, we aim to underline the importance of performing surgery when the clinical condition of the patient is sufficiently stable to be undergone.

The particular clinical condition of our patient, in addition to the necessity for a shorter operative time, the risk of the hostile operative field, and a higher conversion rate from laparoscopic to open procedure in the setting of CCF, made the laparotomic system safer than the laparoscopic approach. In the end, cholecystectomy with the fistula tract resection should be recommended for that medical case.

## Conclusions

Gallbladder perforation with a colic fistula is often a rare complication of chronic gallbladder disease. Symptoms are minimal and usually nonspecific, and preoperative instrumental investigations often fail the diagnosis, which is considerably challenging for the surgeons.

We hope that outlining this case will encourage the report of further instances to develop guidelines for an optimal diagnosis and surgical management of this rare entity.

## Patient consent

Written informed consent was obtained from the patient.
